# Supplementary material for: Rubisco deactivation and chloroplast electron transport rates co-limit photosynthesis above optimal leaf temperature in terrestrial plants
Source: Nat Commun. 2023 May 17;14:2820. doi: 10.1038/s41467-023-38496-4 (PMC10192301; doi:10.1038/s41467-023-38496-4)
Supplement: Supplementary file 1 — Supplementary Information New [file 41467_2023_38496_MOESM1_ESM.pdf]

Supplementary Table 1. Rubisco kinetic,activation state, and chloroplast electron transport rate (J) parameters used in species independent predictions of the temperature response of photosynthetic net CO_2 assimilation (A_n). Cool or warm distinguishes whether the parameter was derived from plants grown at a day growth temperature below or above 25°C, respectively.

Parameter	Source	Definition	Unit	Value at 25°C	Ea (kJ Mol^{-1})	Constant
n	Onoda et al. 2017[1]	Number of Rubisco catalytic sites	µmol m^{-2}			25.6
k_{cat} - cool	Orr et al. 2016[2]; Galmés et al. 2016[3] (in vitro)	The rate constant of Rubisco CO_2 fixation	CO_2 s^{-1}	3.19	61.2	
k_{cat} - warm	Orr et al. 2016; Galmés et al. 2016 (in vitro)	The rate constant of Rubisco CO_2 fixation	CO_2 s^{-1}	2.57	69.3	
K_c - cool	Orr et al. 2016; Galmés et al. 2016 (in vitro)	Michaelis-Menten constant of Rubisco for CO_2	Pa	40.9	58.0	
K_c - warm	Orr et al. 2016; Galmés et al. 2016 (in vitro and in vivo)	Michaelis-Menten constant of Rubisco for CO_2	Pa	35.9	57.3	
K_o - cool	Orr et al. 2016; Galmés et al. 2016 (in vitro and in vivo)	Michaelis-Menten constant of Rubisco for O_2	kPa	29.2	27.4	
K_o - warm	Orr et al. 2016; Galmés et al. 2016 (in vitro and in vivo)	Michaelis-Menten constant of Rubisco for O_2	kPa	30.3	7.26	
Γ^* - cool	Orr et al. 2016; Galmés et al. 2016 (in vitro and in vivo)	CO_2 compensation point in the absence of mitochondrial respiration	Pa	3.94	26.7	
Γ^* - warm	Orr et al. 2016; Galmés et al. 2016 (in vitro and in vivo)	CO_2 compensation point in the absence of mitochondrial respiration	Pa	3.91	30.0	
E_d - cool	iteration of cool climate Rubisco activation state	Deactivation energy	kJ mol^{-1}			199
E_d - warm	iteration of warm climate Rubisco activation state	Deactivation energy	kJ mol^{-1}			212
$T_{0.5}$ - cool	iteration of cool climate Rubisco activation state	Temperature at which enzymatic activity is halved	Kelvin			312
$T_{0.5}$ - warm	iteration of warm climate Rubisco activation state	Temperature at which enzymatic activity is halved	Kelvin			316
$J(To)$ -cool	June et al. 2004[4] equation applied to J of cool grown plants	Electron transport rate at its temperature optimum	µmol e m^{-2} s^{-1}			190
$J(T_o)$ - warm	June et al. 2004[4] equation applied to J of warm grown plants	Electron transport rate at its temperature optimum	µmol e m^{-2} s^{-1}			242
T_o - cool	June et al. 2004[4] equation applied to J of cool grown plants	The temperature optimum of J	°C			31.7
T_o - warm	June et al. 2004[4] equation applied to J of warm grown plants	The temperature optimum of J	°C			36.9
Ω - cool	June et al. 2004[4] equation applied to J of cool grown plants	Difference in temperature from T_o at which J declines to e^{-1} (0.37)	°C			20.1
Ω - warm	June et al. 2004[4] equation applied to J of warm grown plants	Difference in temperature from T_o at which J declines to e^{-1} (0.37)	°C			21.3

[1] Onoda Y, Wright IJ, Evans JR, Hikosaka K, Kitajima K, Niinemets Ü, et al. Physiological and structural tradeoffs underlying the leaf economics spectrum. New Phytol 2017, 214(4): 1447-1463.

[2] Orr, D. J. et al. Surveying Rubisco diversity and temperature response to improve crop photosynthetic efficiency. Plant Physiol. 172, 707-717, doi:10.1104/pp.16.00750 (2016).

[3] Galmés, J., Hermida-Carrera, C., Laanisto, L. & Niinemets, Ü. A compendium of temperature responses of Rubisco kinetic traits: variability among and within photosynthetic groups and impacts on photosynthesis modeling. J. Exp. Bot. 67, 5067-5091, doi:DOI: 10.1093/jxb/erw267 (2016).

[4] June T, Evans JR, Farquhar GD. A simple new equation for the reversible temperature dependence of photosynthetic electron transport: a study on soybean leaf. Funct Plant Biol , 31(3): 275-283. doi:10.1071/FP03250 (2004)

Supplementary Table 2. Metadata relating to published Rubisco activation state observations. Climate distinguishes whether the species was grown at a day growth temperature below (cool) or above (warm) 25°C. Where plants were grown at 25°C, cool or warm grown distinction was made based on whether the peak in photosynthesis was below or above 30°C, respectively.

Species	Climate	Plant Functional Type	Day Growth temperature	Reference	DOIs
Arabidopsis thaliana	cool	herb/grass	21; 23	Carmo-Silva & Salvucci 2011[#]; Kim & Portis 2005; Salvucci et al. 2006	10.1007/s11120-011-9667-8; 10.1093/pcp/pci052; 10.1093/jxb/erl140
Camelina sativa	cool	herb/grass	23; 25	Carmo-Silva & Salvucci 2011; Carmo-Silva & Salvucci 2012	10.1007/s11120-011-9667-8; 10.1007/s00425-012-1691-1
Deschampsia antarctica	cool	herb/grass	12	Salvucci & Crafts-Brandner 2004	10.1104/pp.103.038323
Gossypium hirsutum	warm	herb/grass	25; 28	Carmo-Silva & Salvucci 2011; Feller et al. 1998; Law & Crafts-Brandner 1999; Crafts-Brandner & Salvucci 2000; Salvucci & Crafts-Brandner 2004; Crafts-Brandner & Law 2000	10.1007/s11120-011-9667-8; 10.1104/pp.116.2.539; 10.1104/pp.120.1.173; 10.1073/pnas.230451497; 10.1111/j.1399-3054.2004.00419.x; 10.1007/s004250000364
Ipomoea batatas	warm	herb/grass	27	Cen & Sage 2005	10.1104/pp.105.066233
Larrea tridentata	warm	shrub	28	Salvucci & Crafts-Brandner 2004	10.1104/pp.103.038323
Nicotiana rusticum	warm	herb/grass	25	Crafts-Brandner & Salvucci 2000	10.1073/pnas.230451497
Nicotiana tobacum	warm	herb/grass	28; 30	Carmo-Silva & Salvucci 2011; Carmo-Silva & Salvucci 2012; Kubien & Sage 2008; Yamori & von Caemmerer 2009	10.1007/s11120-011-9667-8; 10.1007/s00425-012-1691-1; 10.1111/j.1365-3040.2008.01778.x; 10.1104/pp.109.146514
Oryza sativa	warm	herb/grass	26; 28	Makino & Sage 2007; Scafaro et al. 2012	10.1093/pcp/pcm118; 10.1111/j.1399-3054.2012.01597.x
Pisum sativum	cool	herb/grass	25	Haldimann & Feller 2005	10.1111/j.1365-3040.2005.01289.x
Populus balsamifera	warm	temperate tree	25	Hozain et al. 2009	10.1093/treephys/tpp091
Populus deltoides	warm	temperate tree	25	Hozain et al. 2009	10.1093/treephys/tpp091
Quercus pubescens	cool	temperate tree	25	Haldimann & Feller 2004	10.1111/j.1365-3040.2004.01222.x
Spinacia Oleracea	cool	herb/grass	15	Yamori et al. 2006	10.1111/j.1365-3040.2006.01550.x
Triticum aestivum	cool	herb/grass	25	Law & Crafts-Brandner 1999	10.1104/pp.120.1.173
Vitis amurensis	warm	shrub	25	Luo et al. 2011	10.1371/journal.pone.0023033
Zea mays	warm	herb/grass	28	Crafts-Brandner & Salvucci 2002	10.1104/pp.002170

[#] *Carmo-Silva & Salvucci 2011 (DOI:10.1007/s11120-011-9667-8) is based on Rubisco activation activity rather than Rubisco activation state perse. We have treated them as equivalent in the context of Rubisco deactivation*

Supplementary Table 3. Metadata relating to published maximum chloroplast electron transport rate (*J*) or quantum efficeincy of PSII (ϕPSII) temperature response curves. *J* was estimated by chlorophyll fluorescence or gas-exchange. T_o is the temperature optimum of *J* (°C), $J(T_o)$ is *J* at the temperature optimum (μmol e m^{-2} s^{-1}), and Ω is the difference in temperature from T_o at which *J* declines to e^{-1} (0.37) (°C). Missing $J(T_o)$ values are where curves were relativised and therefore the parameter was not obtained, including when curves where based on ϕPSII and $J(T_o)$ is not applicable. Climate distinguishes whether the species was grown at a day growth temperature below (cool) or above (warm) 25°C. Where plants were grown at 25°C, cool or warm grown distinction was made based on whether T_o was below or above 30°C, respectively.

Species	Climate	Plant Functional Type	Day Growth	Reference	DOIs	parameter	T_o	$J(T_o)$	Ω
Abutilon theophrasti	cool	herb/grass	16	Ziska 2001; June et al. 2004	10.1034/j.1399-3054.2001.1110309.x; 10.1071/FP03250	J	28.4	202	22.3
Acer pseudoplatanus	cool	temperate tree	22	Dreyer et al. 2001; June et al. 2004	10.1093/treephys/21.4.223; 10.1071/FP03250	J	30.6	167.8	16.9
Betula pendula	cool	temperate tree	22	Dreyer et al. 2001; June et al. 2004	10.1093/treephys/21.4.223; 10.1071/FP03250	J	35.9	161.7	21.6
Betula pendula	cool	temperate tree	20	Wittmann & Pfanz 2007	10.1093/jxb/erm313	ϕPSII	27.2		25.4
Eucalyptus pauciflora	warm	temperate tree	25	Kirschbaum & Farquhar 1984; June et al. 2004	10.1071/PP9840519; 10.1071/FP03250	J	31.8		22
Fagus sylvatica	cool	temperate tree	22	Dreyer et al. 2001; June et al. 2004	10.1093/treephys/21.4.223; 10.1071/FP03250	J	34.7	166.9	18.7
Fagus sylvatica	cool	temperate tree	20	Wittmann & Pfanz 2007	10.1093/jxb/erm313	ϕPSII	36.1		32.2
Fraxinus excelsior	cool	temperate tree	22	Dreyer et al. 2001; June et al. 2004	10.1093/treephys/21.4.223; 10.1071/FP03250	J	32.1	189	16.7
Glycine max	warm	herb/grass	32	Harley et al. 1992; June et al. 2004	10.1111/j.1365-3040.1992.tb00974.x; 10.1071/FP03250	J	39.3	219	19.8
Ipomoea batatas	warm	herb/grass	27	Cen & Sage 2005	10.1104/pp.105.066233	J	30.6	351	14.9
Juglans regia	cool	temperate tree	22	Dreyer et al. 2001; June et al. 2004	10.1093/treephys/21.4.223; 10.1071/FP03250	J	35.7	143.2	18.5
Lycopersicon esculentum	warm	herb/grass	25	Havaux 1992	10.1104/pp.100.1.424	ϕPSII	30.3		12.3
Musa sp.	warm	herb/grass	30	Dongsansuk & Neüner 2013	10.1007/s11099-012-0070-2	ϕPSII	29.4		26.2
Narcissus cyclamineus	cool	herb/grass	20	Dongsansuk & Neüner 2013	10.1007/s11099-012-0070-2	ϕPSII	22.8		25.8
Nicotiana tobacum	warm	herb/grass	30	Yamori et al. 2011	10.1104/pp.110.168435	J	32.1	177	18.6
Nicotinia tobacum	warm	herb/grass	25	Bernacchi et al. 2003; June et al. 2004	10.1046/j.0016-8025.2003.01050.x; 10.1071/FP03250	J	55.6	408	30.2
Oryza sativa	warm	herb/grass	26	Makino and Sage 2007	10.1093/pcp/pcm118	ϕPSII	34.2		21.3
Pinus radiata	cool	needle-leaf tree	24	Walcroft et al. 1997; June et al. 2004	10.1046/J.1365-3040.1997.D01-31.X; 10.1071/FP03250	J	34.6	63.1	23.9
Pinus sylvestris	cool	needle-leaf tree	20	Wang et al. 1996; June et al. 2004	10.1016/0168-1923(96)02329-5	J	21.9	155.7	14.3
Plantago asiatica	cool	herb/grass	15	Hikosaka et al 2005	10.1093/jxb/erj049	J	32.6	203	21.8
Plantago asiatica	warm	herb/grass	30	Hikosaka et al 2005	10.1093/jxb/erj049	J	37.2	209	22.6
Populus tremula	cool	temperate tree	15	Niinemets et al. 1999; June et al. 2004	10.1046/j.1365-3040.1999.00510.x; 10.1071/FP0325	J	35.6	191.4	12.5
Populus tremula	cool	temperate tree	17	Huve et al 2006	10.1111/j.1365-3040.2005.01414.x	J	31.9		17
Prunus persica	warm	temperate tree	29	Walcroft et al. 2002; June et al. 2004	10.1093/treephys/22.13.929; 10.1071/FP03250	J	40	175	23.6
Quercus petraea	cool	temperate tree	22	Dreyer et al. 2001; June et al. 2004	10.1093/treephys/21.4.223; 10.1071/FP03250	J	39.4	231.6	22.5
Quercus robur	cool	temperate tree	22	Dreyer et al. 2001; June et al. 2004	10.1093/treephys/21.4.223; 10.1071/FP03250	J	40.6	226.3	25.8
Solanum tuberosum	cool	herb/grass	25	Havaux 1992	10.1104/pp.100.1.424	ϕPSII	25.2		12.9
Spinacia Oleracea	cool	herb/grass	15	Yamori et al 2008	10.1093/pcp/pcn030	J	29.4	195	17
Spinacia Oleracea	warm	herb/grass	30	Yamori et al 2008	10.1093/pcp/pcn030	J	36.4	158	20.1
Tilia cordata	cool	temperate tree	15	Niinemets et al. 1999; June et al. 2004	10.1046/j.1365-3040.1999.00510.x; 10.1071/FP0325	J	39.2	52.2	16.9
Triticum aestivum	cool	herb/grass	25	Yamasaki et al. 2002	10.1104/pp.010919	J	22.8	519	19.8
Triticum aestivum	cool	herb/grass	25	Silva-Pérez et al. 2017	10.1111/pce.12953	J	29.6	179	26.9
Vitis vinifera	warm	shrub	27	Greer 2022	10.1071/FP21331	ϕPSII	38.6		14.6

Supplementary Table 4. Metadata relating to published photosynthesis temperature response curves and model outcomes. Climate distinguishes whether the species was grown at a day growth temperature below (cool) or above (warm) 25°C. Where plants were grown at 25°C, cool or warm grown distinction was made based on whether the peak in photosynthesis was below or above 30°C, respectively. *mn* is the modeled number of Rubisco catalytic sites (μmol m^{-2}) and *J* the electron transport rate (μmol e m^{-2} s^{-1}) calculated to match A_n at 22°C. The mean root mean squared error (RMSE) of observed net CO_2 assimilation (A_n) versus predicted carboxylationlimited assimilation (A_c) or RuBP regeneration limited assimilation (A_r) are provided for each temperature response curve.

Species	Climate	Plant Functional Type	Day Growth temperature	*mn*	*J*	RMSE (A_c)	RMSE (A_r)	Reference	DOIs
Arabidopsis thaliana	cool	herb/grass	21	21.6	112.4	1.2	1.9	Kim & Portis 2005	10.1093/pcp/pci052
Atractocarpus fitzalanii	warm	tropical tree	27.5	16.7	94.6	0.8	0.8	Choury et al. 2022	10.1111/nph.18077
Atractocarpus fitzalanii	cool	tropical tree	24	10.6	54.9	2.9	2.6	Choury et al. 2022	10.1111/nph.18077
Atractocarpus fitzalanii	warm	tropical tree	31	15.5	87.8	1.8	1.7	Choury et al. 2022	10.1111/nph.18077
Atriplex glabriuscula	cool	herb/grass	10	31.7	171.2	7.4	8.8	Berry & Bj√∂rkman 1980	10.1146/annurev.pp.31.060180.002423
Backhousia citriodora	warm	tropical tree	27.5	18.5	104.9	1.5	1.2	Choury et al. 2022	10.1111/nph.18077
Backhousia citriodora	warm	tropical tree	27.5	19.5	110.4	3.8	3.7	Choury et al. 2022	10.1111/nph.18077
Calophyllum longifolium	warm	tropical tree	32.6	14.1	80.1	3.8	3.9	Slot & Winter 2017	10.1111/pce.13071
Camelina sativa	cool	herb/grass	25	30.4	154.6	2.3	2.1	Carmo-Silva & Salvucci 2012	10.1007/s00425-012-1691-1
Cryptocarya laevigata	warm	tropical tree	27.5	9.0	50.9	0.4	0.5	Choury et al. 2022	10.1111/nph.18077
Cryptocarya laevigata	cool	tropical tree	24	10.1	52.6	0.4	0.8	Choury et al. 2022	10.1111/nph.18077
Deschampsia antarctica	cool	herb/grass	12	21.7	111.2	6.6	7.9	Salvucci & Crafts-Brandner 2004	10.1104/pp.103.038323
Eucalyptus carnaldulensis	warm	temperate tree	35	17.4	98.8	0.6	1.1	Ferrar et al. 1989	10.1071/PP9890199
Eucalyptus crebra	warm	temperate tree	29	22.3	126.6	6.9	7.1	Yin et al. 2012	10.1093/treephys/tpr141
Eucalyptus globulus	cool	temperate tree	25	22.8	117.7	3.2	3.4	Battaglia et al. 1996	10.1093/treephys/16.1-2.81
Eucalyptus miniata	warm	temperate tree	35	14.4	81.4	1.7	1.7	Ferrar et al. 1989	10.1071/PP9890199
Eucalyptus pauciflora	warm	temperate tree	30	55.3	313.4	4.5	5.6	Hikosaka 1997	10.1006/anbo.1997.0512
Eucalyptus pauciflora	cool	temperate tree	20	49.6	257.9	3.5	2.7	Hikosaka 1997	10.1006/anbo.1997.0512
Eucalyptus regnans	cool	temperate tree	15	15.6	81.5	1.6	1.8	Warren 2008	10.1093/treephys/28.1.11
Eucalyptus regnans	warm	temperate tree	30	18.3	103.9	1.6	2.3	Warren 2008	10.1093/treephys/28.1.11
Eucalyptus tereticornis	cool	temperate tree	18	30.4	156.6	1.9	1.7	Kumarathunge et al 2020	10.1111/gcb.14975
Ficus insipida	warm	tropical tree	32.6	26.6	150.6	3.0	2.7	Slot & Winter 2017	10.1111/pce.13071
Flindersia australis	warm	tropical tree	27.5	16.2	91.9	2.8	2.6	Choury et al. 2022	10.1111/nph.18077
Flindersia australis	warm	tropical tree	31	13.8	78.5	2.6	2.4	Choury et al. 2022	10.1111/nph.18077
Garcinia madruno	warm	tropical tree	32.6	10.4	60.3	0.4	0.4	Slot & Winter 2017	10.1111/pce.13071
Glycine max	warm	herb/grass	35	34.8	199.8	1.7	2.2	Vu et al. 1997	10.1046/j.1365-3040.1997.d01-10.x
Gossypium barbadense	warm	shrub	32	31.5	178.6	3.7	1.9	Wise et al. 2004	10.1111/j.1365-3040.2004.01171.x
Gossypium hirsutum	warm	herb/grass	28	43.1	247.8	3.0	6.2	Law & Crafts-Brandner 1999	10.1104/pp.120.1.173
Gossypium hirsutum	warm	herb/grass	28	32.6	187.7	4.0	7.3	Crafts-Bradner & Law 2000	10.1007/s004250000364
Gossypium hirsutum	warm	herb/grass	25	41.8	239.9	3.2	5.8	Crafts-Bradner & Salvucci 2000	10.1073/pnas.230451497
Harungana montana	cool	tropical montane	21.5	16.9	87.9	2.8	3.3	Dusenge et al. 2021	10.1111/gcb.15790
Harungana montana	warm	tropical montane	27	13.6	76.8	2.3	2.4	Dusenge et al. 2021	10.1111/gcb.15790
Harungana montana	warm	tropical montane	29.6	12.8	72.6	2.5	2.7	Dusenge et al. 2021	10.1111/gcb.15790
Ipomoea batatas	warm	herb/grass	27	23.0	130.6	1.6	1.6	Cen & Sage 2005	10.1104/pp.105.066233
Lagerstroemia speciosa	warm	tropical tree	32.6	22.0	125.1	2.2	2.4	Slot & Winter 2017	10.1111/pce.13071
Larrea divaricata	warm	shrub	44	26.3	149.3	8.9	6.2	Berry & Bj√∂rkman 1980	10.1146/annurev.pp.31.060180.002423
Larrea tridentate	warm	shrub	28	39.6	226.6	3.4	4.7	Salvucci & Crafts-Brandner 2004	10.1104/pp.103.038323
Nerium oleander	warm	shrub	35	11.3	64.1	1.5	1.5	Ferrar et al. 1989	10.1071/PP9890199
Nicotiana tobacum	warm	herb/grass	28	36.3	206.0	3.1	2.7	Kubien & Sage 2008	10.1111/j.1365-3040.2008.01778.x
Nicotiana tobacum	cool	herb/grass	20	25.9	134.4	3.3	2.6	Yamori & von Caemmerer 2009	10.1104/pp.109.146514
Nicotiana tobacum	warm	herb/grass	27	36.2	205.0	1.7	2.1	Yamori & Makino 2011	10.1111/j.1365-3040.2011.02280.x
Nicotiana tobacum	warm	herb/grass	30	24.2	137.3	0.8	0.5	Yamori & von Caemmerer 2009	10.1104/pp.109.146514
Ochroma pyramidale	warm	tropical tree	33	23.7	134.5	2.8	2.6	Cheesman & Winter 2013	10.1093/jxb/ert211
Ormosia macrocalyx	warm	tropical tree	30	10.9	62.3	2.2	2.1	Cheesman & Winter 2013	10.1093/jxb/ert211
Oryza meridionalis	warm	herb/grass	28	24.4	138.4	1.9	1.8	Scafaro et al. 2012	10.1111/j.1399-3054.2012.01597.x
Oryza sativa	warm	herb/grass	27	39.4	223.1	1.7	0.8	Yamori & Makino 2011	10.1111/j.1365-3040.2011.02280.x
Oryza sativa	warm	herb/grass	28	32.9	186.8	1.0	1.1	Scafaro et al. 2012	10.1111/j.1399-3054.2012.01597.x
Oryza sativa	warm	herb/grass	26	40.9	231.8	3.1	3.6	Makino & Sage 2007	10.1093/pcp/pcm118
Picea mariana	cool	needle-leaf tree	22	10.2	53.0	1.4	1.6	Way & Sage 2008	10.1111/j.1365-3040.2008.01842.x
Picea mariana	warm	needle-leaf tree	30	11.2	63.2	0.9	1.1	Way & Sage 2008	10.1111/j.1365-3040.2008.01842.x
Picea mariana	cool	needle-leaf tree	22	9.8	51.0	1.9	2.2	Way & Sage 2008	10.1111/j.1365-3040.2008.01842.x
Pinus taeda	warm	needle-leaf tree	32	15.7	89.3	3.1	3.2	Yin et al. 2012	10.1093/treephys/tpr141
Pisum sativum	cool	herb/grass	25	12.0	62.4	1.5	0.4	Haldimann & Feller 2005	10.1111/j.1365-3040.2005.01289.x
Plantago asiatica	cool	herb/grass	15	30.1	156.3	1.2	1.5	Hikosaka et al. 2006	10.1093/jxb/erj049
Populus balsmifera	warm	temperate tree	25	23.2	132.1	6.4	6.1	Hozain et al. 2009	10.1093/treephys/tpp091
Populus maximowiczii x Populus balsamifera	cool	temperate tree	23	19.5	101.1	3.9	4.5	Benomar et al. 2019	10.1371/journal.pone.0206021
Populus maximowiczii x Populus balsamifera	warm	temperate tree	33	19.5	110.4	1.1	1.0	Benomar et al. 2019	10.1371/journal.pone.0206021
Populus maximowiczii x Populus nigra	cool	temperate tree	23	15.6	81.0	2.0	2.1	Benomar et al. 2019	10.1371/journal.pone.0206021
Populus maximowiczii x Populus nigra	warm	temperate tree	33	20.6	116.5	0.6	1.0	Benomar et al. 2019	10.1371/journal.pone.0206021
Quercus canariensis	cool	temperate tree	23	21.4	111.5	1.4	1.6	Warren & Dreyer 2006	10.1093/jxb/erl067
Quercus pubescens	cool	temperate tree	25	17.5	91.1	1.1	2.2	Haldimann & Feller 2004	10.1111/j.1365-3040.2004.01222.x
Rubus idaeus	cool	shrub	20	14.1	73.2	2.2	2.1	Choury et al. 2022	10.1111/pce.12997
Sextonia rubra	warm	tropical tree	30	12.8	76.4	0.5	0.8	Doughty & Goulden 2008	10.1029/2007JG000632
Spinacia Oleracea	cool	herb/grass	15	17.8	92.5	3.1	3.6	Yamori et al. 2006	10.1111/j.1365-3040.2006.01550.x
Spinacia Oleracea	warm	herb/grass	27	44.3	251.2	3.5	4.2	Yamori & Makino 2011	10.1111/j.1365-3040.2011.02280.x
Spinacia Oleracea	warm	herb/grass	30	20.0	118.1	3.2	4.0	Yamori & Makino 2011	10.1111/j.1365-3040.2006.01550.x
Syzygium guineense	cool	tropical montane	21.5	12.8	66.4	1.8	2.1	Dusenge et al. 2021	10.1111/gcb.15790
Syzygium guineense	warm	tropical montane	27	13.4	76.1	3.2	3.3	Dusenge et al. 2021	10.1111/gcb.15790
Syzygium guineense	warm	tropical montane	29.6	14.7	83.3	3.2	3.3	Dusenge et al. 2021	10.1111/gcb.15790
Tristaniopsis Laurina	warm	tropical tree	27.5	17.7	100.1	3.9	4.1	Choury et al. 2022	10.1111/nph.18077
Triticum aestivum	cool	herb/grass	25	35.1	182.2	2.7	4.8	Law & Crafts-Brandner 1999	10.1104/pp.120.1.173
Triticum aestivum	warm	herb/grass	27	35.7	202.3	2.8	3.1	Yamori & Makino 2011	10.1111/j.1365-3040.2011.02280.x
Vitis amurensis	warm	shrub	25	13.9	78.6	1.1	1.0	Luo et al. 2011	10.1371/journal.pone.0023033
Xanthostemon chrysanthus	warm	tropical tree	27.5	22.6	127.9	4.7	4.5	Choury et al. 2022	10.1111/nph.18077
Xanthostemon chrysanthus	warm	tropical tree	31	14.7	83.6	2.2	1.8	Choury et al. 2022	10.1111/nph.18077

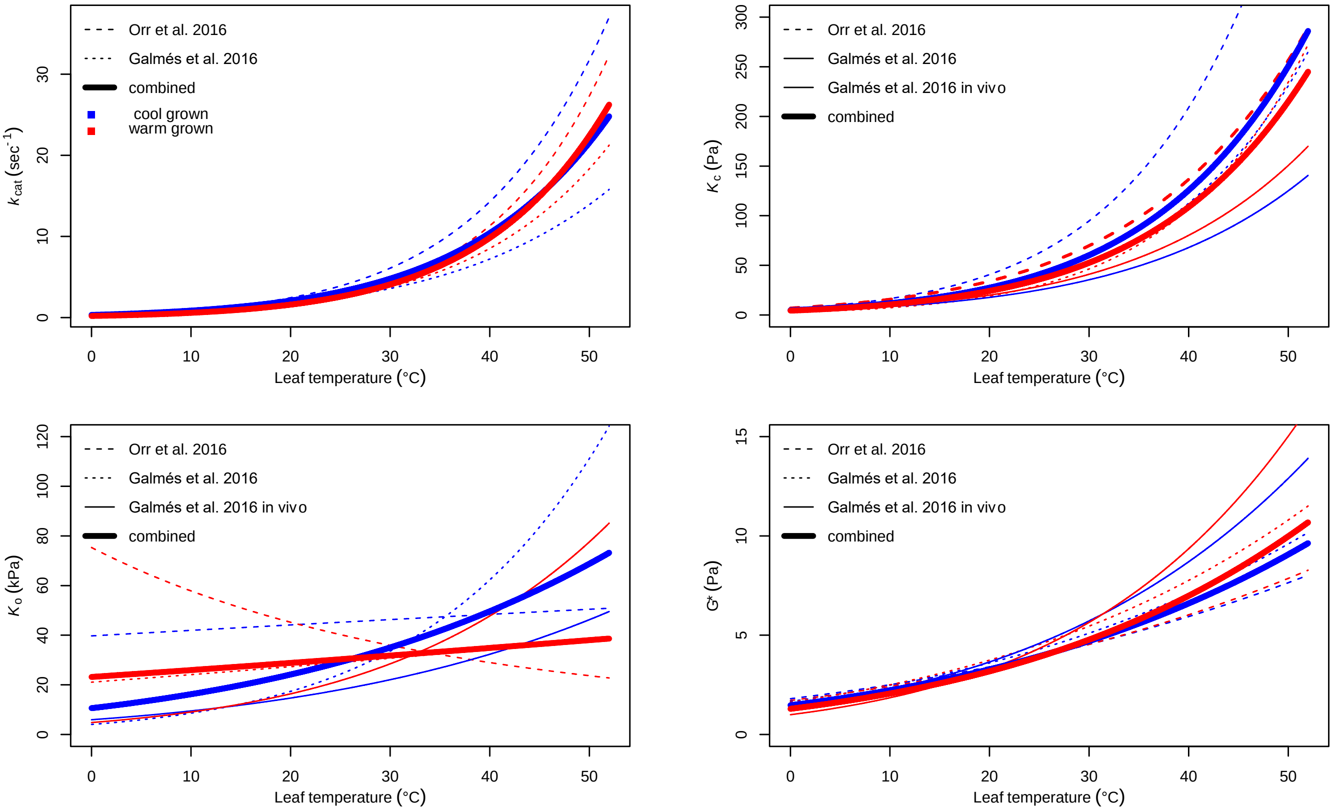

Supplementary Figure 1. The temperature response of Rubisco kinetics from multiple species studies that we have used for FvCB model parameterisation. The rate constant of Rubisco CO_2 fixation (k_{cat}), the Michaelis-Menten constant of Rubisco for CO_2 (K_c), the Michaelis-Menten constant of Rubisco for oxygen (K_o), and the CO_2 compensation point in the absence of light respiration (Γ^*). Refer to Supplementary Table 1 for values at 25°C and the corresponding activation Energy (E_a) that each curve was derived from.

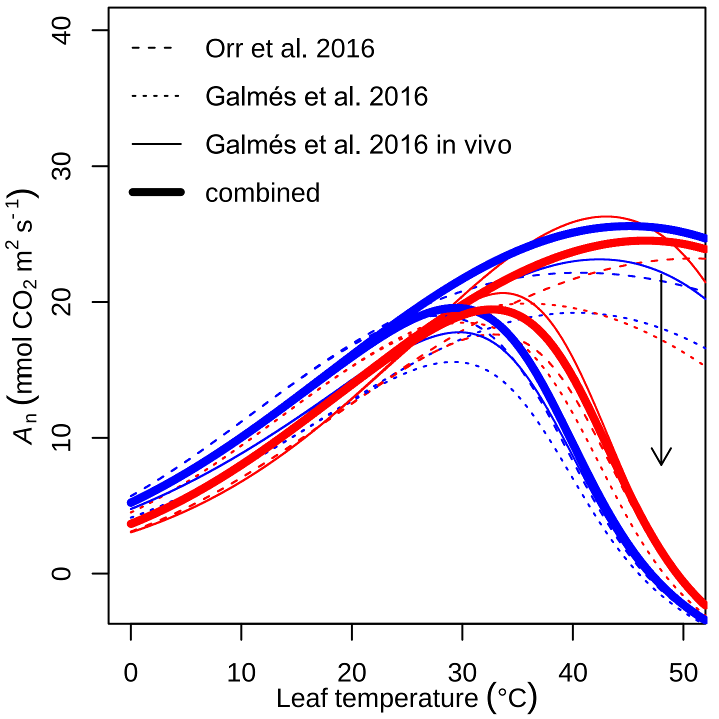

Supplementary Figure 2. The FvCB model was parameterised using either the *in vitro* Rubisco kinetics presented in Orr, et al. [19], the *in vivo* and *in vitro* kinetics presented in Galmés, et al. [18], or the combined mean value of all three. Model simulations were run with and without Rubisco activation included, evident by the distinctive decline in A_n at higher temperatures when activation was included (marked by the arrow).

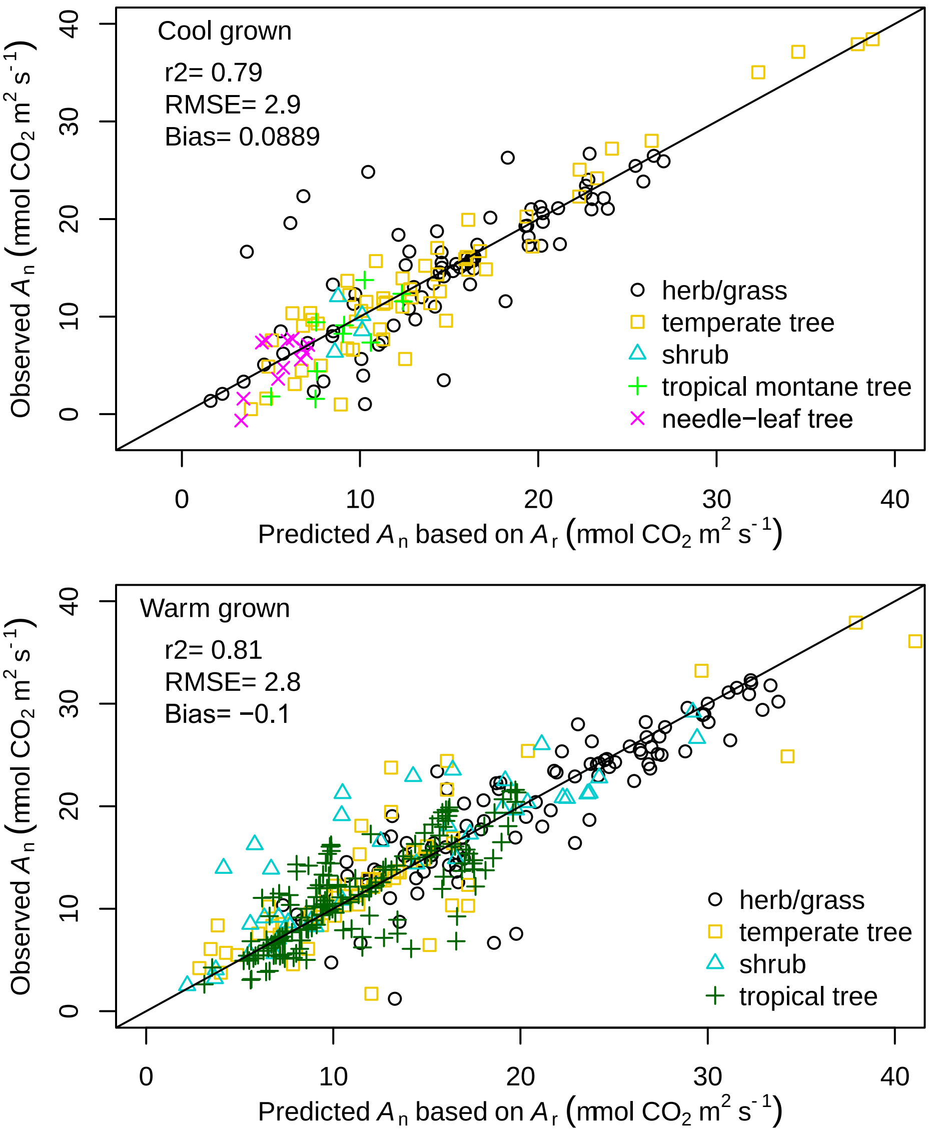

Supplementary Figure 3. Predictions of RuBB regeneration limited net CO_2 assimilation rates (A_r) were plotted against corresponding observations for cool and warm grown plants. The coefficient of determination (r^2), a 1:1 ratio (solid line), the root mean squared error (RMSE) between observed and predicted values, and the bias in observations being greater than predictions for each growth environment are provided. Plant functional types are indicated by differing colours and symbols.

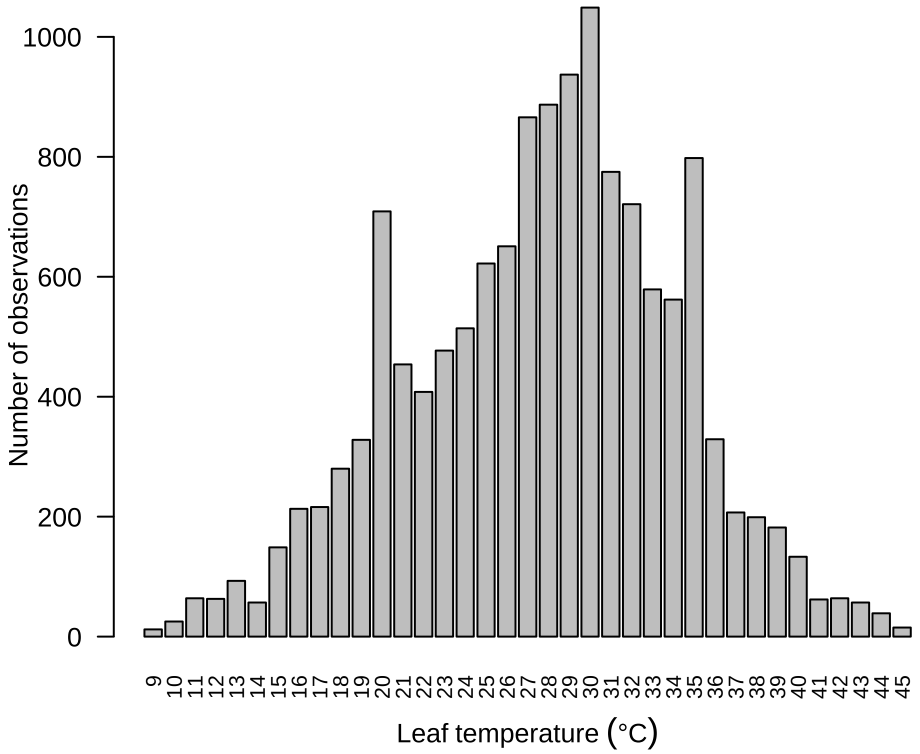

Supplementary Figure 4. The number of net CO_2 assimilation rate (A_n) observations at each leaf temperature when binned per degree Celsius for the global gas-exchange dataset[33] analysed in Fig. 3b.

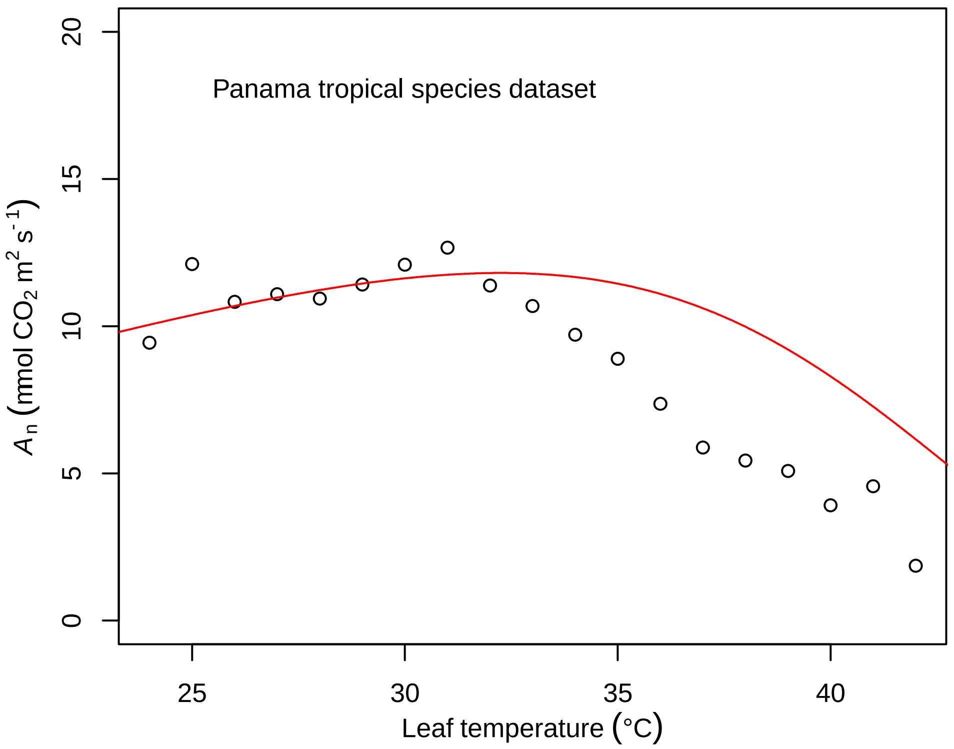

Supplementary Figure 5. The temperature response of net CO_2 assimilation rates (A_n) in tropical rainforest tree and liana species from Panama[12]. Observations (open circles) are the mean A_n values binned per degree Celsius and consisting of 3942 observations from 42 species. The red curve is the predicted temperature response of A_c with Rubisco activation included for warm grown plants. Note that the greater than predicted decline in observed A_n at temperatures above 30°C is consistent with the published observations of declines in stomatal conductance and intercellular CO_2 concentrations limiting photosynthesis in the study from which the data were collected[12].

Supplementary Figure 6. PRISMA flowchart

PRISMA 2020 flow diagram for new systematic reviews which included searches of databases, registers and other sources

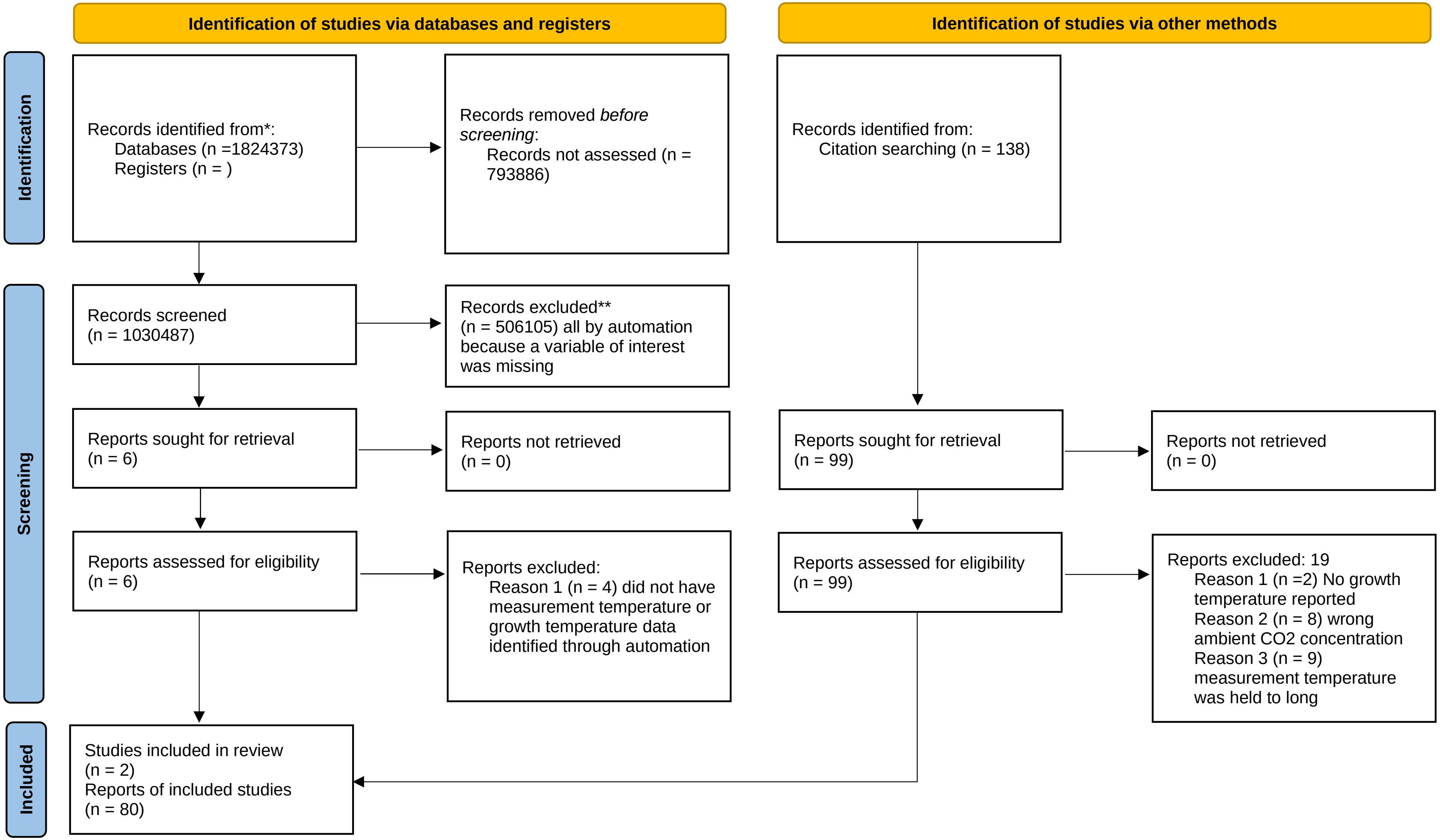

*Consider, if feasible to do so, reporting the number of records identified from each database or register searched (rather than the total number across all databases/registers).
**If automation tools were used, indicate how many records were excluded by a human and how many were excluded by automation tools.

From: Page MJ, McKenzie JE, Bossuyt PM, Boutron I, Hoffmann TC, Mulrow CD, et al. The PRISMA 2020 statement: an updated guideline for reporting systematic reviews. BMJ 2021;372:n71. doi: 10.1136/bmj.n71. For more information, visit: http://www.prisma-statement.org/
