# Supplementary material for: Rubisco deactivation and chloroplast electron transport rates co-limit photosynthesis above optimal leaf temperature in terrestrial plants
Source: Nat Commun. 2023 May 17;14:2820. doi: 10.1038/s41467-023-38496-4 (PMC10192301; doi:10.1038/s41467-023-38496-4)
Supplement: Supplementary file 2 — Reporting Summary [file 41467_2023_38496_MOESM2_ESM.pdf]

nature portfolio

Corresponding author(s): Andrew P Scafaro
Last updated by author(s): Nov 7, 2022

Reporting Summary

Nature Portfolio wishes to improve the reproducibility of the work that we publish. This form provides structure for consistency and transparency in reporting. For further information on Nature Portfolio policies, see our Editorial Policies and the Editorial Policy Checklist.

Statistics

For all statistical analyses, confirm that the following items are present in the figure legend, table legend, main text, or Methods section.

n/a	Confirmed	
x	☐	The exact sample size (n) for each experimental group/condition, given as a discrete number and unit of measurement
x	☐	A statement on whether measurements were taken from distinct samples or whether the same sample was measured repeatedly
☐	x	The statistical test(s) used AND whether they are one- or two-sided<br>*Only common tests should be described solely by name; describe more complex techniques in the Methods section.*
x	☐	A description of all covariates tested
x	☐	A description of any assumptions or corrections, such as tests of normality and adjustment for multiple comparisons
x	☐	A full description of the statistical parameters including central tendency (e.g. means) or other basic estimates (e.g. regression coefficient) AND variation (e.g. standard deviation) or associated estimates of uncertainty (e.g. confidence intervals)
☐	x	For null hypothesis testing, the test statistic (e.g. F, t, r) with confidence intervals, effect sizes, degrees of freedom and P value noted<br>*Give P values as exact values whenever suitable.*
x	☐	For Bayesian analysis, information on the choice of priors and Markov chain Monte Carlo settings
x	☐	For hierarchical and complex designs, identification of the appropriate level for tests and full reporting of outcomes
x	☐	Estimates of effect sizes (e.g. Cohen's d, Pearson's r), indicating how they were calculated

Our web collection on statistics for biologists contains articles on many of the points above.

Software and code

Policy information about availability of computer code

Data collection	R Language for statistical computing and graphics. R version 4.1.2 (2021-11-01)<br>R Package digitize, version 0.0.4<br>https://doi.org/10.5281/zenodo.7683559
Data analysis	R Language for statistical computing and graphics. R version 4.1.2 (2021-11-01)<br>R Package readxl, version 1.3.1<br>R Package Metrics, version 0.1.4<br>https://doi.org/10.5281/zenodo.7683559

For manuscripts utilizing custom algorithms or software that are central to the research but not yet described in published literature, software must be made available to editors and reviewers. We strongly encourage code deposition in a community repository (e.g. GitHub). See the Nature Portfolio guidelines for submitting code & software for further information.

Data

Policy information about availability of data

All manuscripts must include a data availability statement. This statement should provide the following information, where applicable:

- Accession codes, unique identifiers, or web links for publicly available datasets
- A description of any restrictions on data availability
- For clinical datasets or third party data, please ensure that the statement adheres to our policy

The author and DOI information relating to the published articles from which all Rubisco kinetic, Rubisco activation state, net carbon assimilation, and electron transport rate temperature response curve data where analysed are presented in Supplementary Tables 1, 2, 3, and 4. The collated data are provided with the model code (https://doi.org/10.5281/zenodo.7683559). The global individual photosynthesis vs leaf temperature and Panama species datasets are available from the TRY plant trait database http://www.try-db.org/TryWeb/Data.php under the dataset names "Global Leaf Gas Exchange Database (I)" and "Photosynthesis Temperature Response Panama".

Human research participants

Policy information about studies involving human research participants and Sex and Gender in Research.

Reporting on sex and gender	NA
Population characteristics	NA
Recruitment	NA
Ethics oversight	NA

Note that full information on the approval of the study protocol must also be provided in the manuscript.

Field-specific reporting

Please select the one below that is the best fit for your research. If you are not sure, read the appropriate sections before making your selection.

- [x] Life sciences
- [ ] Behavioural & social sciences
- [ ] Ecological, evolutionary & environmental sciences

For a reference copy of the document with all sections, see nature.com/documents/nr-reporting-summary-flat.pdf

Life sciences study design

All studies must disclose on these points even when the disclosure is negative.

Sample size	For Rubisco activation state, all previous publications that contained a temperature response curve of Rubisco activation state or Rubisco activation activity that was found using key search terms was included in the study. This comprised of curves representing 17 species. Published curves were the means of 3 or more biological replications per species. The activation state response to temperature was limited by the number for published studies found. For the temperature response of photosynthesis observations, analysis of 75 previously published photosynthesis temperature response curves was performed. Each published curve was in most instances a mean curve from 3 or more biological replicates. Samples size was chosen based on the availability of temperature response of photosynthesis data available in the literature that stated a clear growth temperature. Further, the photosynthesis model was fit to 13,876 individual photosynthesis observations from 311 species based on a publicly available global plant gas-exchange database. The sample size is sufficient because it covers C3 plant species of all major functional type (from desert shrubs to alpine conifers) and thus gives a broad representation of the global pattern of the response of photosynthesis to temperature.
Data exclusions	If there were multiple photosynthesis temperature curves in a study , only the curve that was closest to the day growth temperature that corresponded to that species optimal growth as outlined in the paper from which the information was extracted was used. When the species was acclimated to extremes the extreme growth temperature was excluded. For example, if spinach was grown at an optimal day temperature of 20°C and a heat growth temperature of 35°C in a study, only the photosynthesis temperature response curve relating to the 20°C growth temperature was used in this analysis
Replication	As this study is an analysis of previously obtained and published data, there was no experiment to replicate. Replication is acquired through intrinsic replication in the published studies from which data is acquired. Where a root mean squared error analysis of predicted versus observed photosynthesis values was performed on individual species photosynthesis temperature response curves the replication is based on individual observations across the entire curve, which ranged from 4 to 38 observations.
Randomization	Data was split between cool and warm grown climates based on a species growing below or above 25°C. For Rubisco activation state and photosynthesis observation data where species were grown at 25°C, if the maximum rate of photosynthesis occurred below or above 30°C it was classified as cool or warm climate, respectively.

Blinding	Data was blind allocated through code software to cool or warm climate groups based on the criteria above.

Reporting for specific materials, systems and methods

We require information from authors about some types of materials, experimental systems and methods used in many studies. Here, indicate whether each material, system or method listed is relevant to your study. If you are not sure if a list item applies to your research, read the appropriate section before selecting a response.

Materials & experimental systems

n/a	Involved in the study
☒	☐ Antibodies
☒	☐ Eukaryotic cell lines
☒	☐ Palaeontology and archaeology
☒	☐ Animals and other organisms
☒	☐ Clinical data
☒	☐ Dual use research of concern

Methods

n/a	Involved in the study
☒	☐ ChIP-seq
☒	☐ Flow cytometry
☒	☐ MRI-based neuroimaging
